# Electronic Nicotine Delivery Systems and Cardiovascular Health: A Narrative Review of E-Cigarettes, Heated Tobacco Products, and the Challenge of Disentangling Risk

**DOI:** 10.7759/cureus.107443

**Published:** 2026-04-21

**Authors:** Elias Nabhan, Alexandrina-Paula Vana, Edouard Naoum Nehme, Rida Khouri, Nabil Poulos

**Affiliations:** 1 Cardiology, Centre Hospitalier Josephine Baker, Gonesse, FRA

**Keywords:** cardiovascular disease, electronic cigarettes, endothelial function, ends, heated tobacco products, iqos, nicotine

## Abstract

Electronic nicotine delivery systems (ENDS), encompassing e-cigarettes, pod devices, and heated tobacco products (HTPs), have attained considerable global prevalence in recent years. While the cardiovascular consequences of conventional tobacco smoking are well characterized, the corresponding risks associated with ENDS remain incompletely understood. This review synthesizes the available evidence across device categories, drawing upon prospective cohort studies, randomized controlled trials (RCTs) of acute physiological effects, and experimental mechanistic research. Current evidence does not allow reliable quantification of cardiovascular risk attributable specifically to ENDS, independent of prior smoking, nor does it support the conclusion that these products are cardiovascularly safe. Dual use is associated with the highest observed risk, though this likely reflects both continued smoking exposure and differences in underlying user characteristics and should not be assumed to confer harm reduction. Direct ENDS versus cigarette comparisons are non-significant and of very low certainty; apparent benefit may reflect smoking cessation rather than a protective effect of ENDS per se. A main evidence gap is the absence of long-duration cohort data among individuals who have never smoked.

## Introduction and background

Cardiovascular disease (CVD) accounts for approximately 18 million deaths annually, with an estimated 523 million patients [1]. Tobacco smoking contributes to this burden through multiple mechanisms, including endothelial injury, oxidative stress, systemic inflammation, and hypercoagulability. Despite numerous public health interventions over several decades, at least 1.2 billion people worldwide were still smoking tobacco products in 2025 [2,3]. E-cigarettes emerged in the mid-2000s, offering a mechanism of nicotine delivery that circumvents combustion and, theoretically, avoids many of the cardiovascular harms associated with it. Whether this hypothesis is supported by the accumulated evidence is the central question this review addresses, approached with appropriate caution regarding the extent to which current data can support definitive conclusions.

The term "Electronic Nicotine Delivery Systems" (ENDS) as used in this review encompasses two different categories. E-cigarettes, including early cigalike devices, refillable tank systems, and more recent high-nicotine pod systems such as JUUL, aerosolize a liquid comprising propylene glycol, vegetable glycerin, flavoring compounds, and variable concentrations of nicotine at temperatures well below the threshold for combustion [4,5]. Heated tobacco products (HTPs), including I Quit Ordinary Smoking (IQOS), glo, and Ploom, represent a different technology: processed tobacco is heated to approximately 350 degrees Celsius without ignition, yielding an aerosol that contains fewer combustion-derived toxicants than cigarette smoke but higher levels of certain toxicants than e-cigarette aerosol, though profiles vary by compound and device type [6]. The two categories share nicotine delivery as a core feature but differ in their chemical profiles and in the demographic characteristics of their typical users. Nicotine activates the sympathetic nervous system, impairs endothelial nitric oxide bioavailability, and promotes platelet aggregation [7]. ENDS aerosols additionally contain reactive oxygen species, fine particulate matter, carbonyl compounds, heavy metals, and nitrosamines, all of which carry established cardiovascular toxicity [8]. Concurrently, the 2024 Cochrane update concluded with high certainty that nicotine-containing e-cigarettes increase long-term abstinence from conventional smoking relative to nicotine replacement therapy, with a relative risk of 1.55 [9].

Published meta-analyses have estimated the adjusted OR for composite CVD in ENDS users at approximately 1.3 relative to never-users, with higher estimates among dual users [10,11]. Direct comparisons of e-cigarettes with conventional cigarettes have not reached statistical significance [12]. These figures are informative but are undermined by a structural limitation pervasive throughout observational literature: the majority of studies cannot distinguish never-smoking ENDS users from former smokers who have transitioned from combustible tobacco. Former smokers retain residual cardiovascular risk (CVR) for years following cessation, and combining them with true never-users yields risk estimates that partly reflect prior tobacco exposure rather than ENDS use per se.

The aim of this review is to describe the current evidence on the cardiovascular effects of electronic nicotine delivery systems (ENDS), including e-cigarettes and heated tobacco products.

## Review

Review approach

Studies were identified through PubMed/MEDLINE, Scopus, CINAHL, Web of Science, Cochrane CENTRAL, and Clinical Trials, covering publications from January 2010 through March 2026. Search terms included combinations of “e-cigarette,” “electronic nicotine delivery systems,” “heated tobacco products,” “IQOS,” “cardiovascular disease,” “myocardial infarction,” “stroke,” “endothelial function,” and “arterial stiffness.” Reference lists of relevant reviews and included studies were also screened to identify additional sources. 

This is a purposive narrative review. Studies were selected based on relevance to cardiovascular outcomes or mechanisms, prioritizing prospective cohort studies for long-term endpoints and randomized controlled trials (RCTs) for acute physiological effects. Cross-sectional studies were included for completeness but interpreted with caution due to inherent limitations in causal inference. No formal systematic review protocol or prespecified inclusion criteria were applied. Given the narrative design, no formal risk-of-bias tool or quantitative synthesis was applied; instead, study quality and certainty were assessed qualitatively based on design, consistency, and susceptibility to confounding.

Previously published systematic reviews and meta-analyses are used as background references rather than primary data sources. The certainty ratings are qualitative, based on factors such as the design of the study, reproducibility between studies, accuracy of the results, and bias risks. These ratings do not reflect formal GRADE scoring; they are descriptive characterizations intended to convey the degree of confidence that the available evidence truly represents.

The confounding problem

Most observational datasets classify participants as "exclusive e-cigarette users" without adequately distinguishing true never-smokers from former smokers who have transitioned to ENDS. Former smokers carry residual CVR that may persist for a decade or more, independent of their current product use [10]. When these individuals are compared with lifetime never-users of any tobacco product, the resulting odds ratio (OR) conflates any potential ENDS-attributable effect with the legacy of prior smoking exposure. The addition of a binary "former smoker yes/no" covariate does not resolve this problem, as the self-reported history of smoking is imprecise, pack-year exposure is rarely captured in sufficient detail, and the cardiovascular consequences of prior smoking cannot be expressed by a single dichotomous variable [10,12]. The clearest evidence of this bias derives from the limited number of studies that restricted analysis to verified never-smoking ENDS users. In this subgroup, composite CVD risk estimates decrease to approximately OR 1.1 and no longer reach statistical significance [13]. The attenuation from the 1.2 to 1.3 range observed in the ENDS user literature to approximately 1.1 in never-smokers indicates that a significant proportion of the apparent risk in larger analyses reflects a residual effect from prior tobacco exposure rather than a direct ENDS effect [11,13]. This does not imply that the independent risk attributable to ENDS is zero. The never-smoker subgroup remains too small to support that conclusion. A modest true independent risk may exist, but it is likely smaller than unadjusted estimates suggest, and its actual magnitude remains undetermined.

Composite CVD and major adverse cardiovascular events (MACE)

Across prospective and retrospective cohort studies, e-cigarette use is associated with elevated rates of composite cardiovascular events and MACE relative to never-use, with modest elevations (often in the range of OR 1.2 to 1.5, though heterogeneous across studies) for composite CVD and MACE [10,11]. These associations are statistically significant, though they fall well below the 2.0 to 4.0 range typically observed for conventional cigarette smoking [12,13].

Dual use represents a categorically distinct risk exposure. Estimates for dual users are higher than for exclusive users, though these findings are influenced by continued smoking exposure and underlying differences in user characteristics [10-13]. This estimate carries greater credibility than exclusive-user figures, as current conventional smoking is present, limiting the impact of confounding by prior tobacco exposure.

The direct e-cigarette versus cigarette comparison is frequently misinterpreted. Pooled OR across available studies is approximately 0.8 to 0.9, with confidence intervals that cross unity and substantial between-study variability [12]. This is directionally consistent with a harm reduction gradient for people who achieve complete switching, but is insufficiently supported by the evidence. The attendant uncertainty is significant and should not be construed as endorsement of ENDS as a cardiovascularly safe alternative.

Myocardial infarction (MI) and coronary artery disease (CAD)

Coronary artery disease (CAD) exhibits the most consistent signal among individual endpoints, with OR broadly approximating 1.2 across cohort studies [13,14]. This finding is mechanistically plausible, given that the hemodynamic and thrombogenic effects most clearly demonstrated in experimental work are directly relevant to coronary pathology [12,13]. Myocardial infarction (MI) data from large surveys and longitudinal studies, including the Population Assessment of Tobacco and Health (PATH) cohort, demonstrate comparable modest elevations [15].

Stroke

The stroke evidence presents a different pattern. Several large cohort studies have failed to identify a statistically significant association following covariate adjustment, and pooled estimates do not reach significance [14]. Whether this reflects pathophysiological differences, in that ENDS may be more relevant to thrombotic coronary events than to cerebrovascular disease, or simply insufficient statistical power given the lower incidence of stroke relative to composite CVD endpoints, remains unclear. The current evidence does not support a confident conclusion regarding ENDS-associated stroke risk [13,14].

Cardiovascular mortality

Very few cohort studies have accumulated adequate follow-up duration to examine cardiovascular mortality as an outcome. Available data indicate hazard ratios of approximately 1.1 for e-cigarette users relative to never-users, a nonsignificant finding [11-14]. This likely reflects insufficient follow-up rather than a true null effect.

Heated tobacco products

The evidence base for HTPs is limited, and this review's treatment of HTPs is limited because available data are insufficient to allow a more extensive discussion. Virtually all research classified as HTP cardiovascular evidence pertains specifically to IQOS. Glo and Ploom are underrepresented in the research record, and findings from IQOS studies may not generalize to other HTP designs that differ in heating element configuration, tobacco composition, and aerosol chemistry [6,15]. 

Long-term clinical outcome data for HTPs are sparse, cross-sectional, or short in follow-up duration and subject to the same smoking history confounding as the e-cigarette literature, especially since IQOS adoption has occurred predominantly among established smokers [13-15]. Directional associations with CVR are reported, with OR in the 1.2 to 1.3 range, but certainty is very low [15]. These findings are preliminary signals rather than reliable effect estimates. The acute experimental evidence for IQOS is more developed. Single-session randomized crossover studies demonstrate significant arterial stiffness increases, with pulse wave velocity (PWV) elevated by approximately 0.7 to 0.9 m/s and platelet thrombus formation comparable to that observed with nicotine-containing e-cigarettes [6,14]. Head-to-head comparisons of IQOS against e-cigarettes show similar acute vascular and hemodynamic effects, with no statistically significant difference in augmentation index between devices [6]. Both differ from conventional cigarettes in the absence of combustion products rather than in nicotine-mediated acute cardiovascular effects [5].

IQOS generates fewer combustion-derived compounds, including less carbon monoxide, benzene, and polycyclic aromatic hydrocarbons, while retaining nicotine delivery and generating a range of carbonyl compounds during the heating process [4-6]. The implications of this intermediate toxicant profile for long-term CVR are unknown. There is no basis for concluding that HTPs are cardiovascularly safe, nor is there adequate evidence to quantify where their risk falls relative to e-cigarettes or conventional cigarettes. Direct comparative CVR between HTPs and e-cigarettes remains essentially unquantified in epidemiological data [14,15].

In addition, emerging device types and newer generations of HTPs remain underrepresented in the current literature. Most available data pertain to earlier iterations of IQOS, and findings may not generalize to evolving products with different heating mechanisms, aerosol composition, and nicotine delivery profiles. This further limits the applicability of existing evidence to current patterns of use.

Scope and limits of RCT

The acute trial literature consists almost entirely of crossover designs with small samples, typically 20 to 120 participants, measuring outcomes within hours of a single-use session. Within these constraints, this is a robust design for the specific question it addresses: randomization, sham conditions, and within-subject comparisons eliminate many of the confounding factors. The limitation is that this design addresses a narrow and specific question: what happens to a physiological parameter in the minutes to hours following a single ENDS-use episode. It cannot address whether that effect accumulates into structural cardiovascular damage over years of daily use. These are distinct questions requiring distinct study designs, and conflating them is a persistent source of misinterpretation in how ENDS trial data are applied.

Hemodynamic effects

Acute nicotine-containing e-cigarette use increases systolic blood pressure (BP) by approximately 10 to 13 mmHg above sham conditions, diastolic BP by approximately 5 mmHg, and heart rate (HR) by 10 to 11 beats per minute [16]. These effects are consistent, reproducible, and nicotine-dependent: nicotine-free devices do not produce them. In direct comparisons with conventional cigarettes, the HR response to e-cigarettes is modestly attenuated, while BP responses are similar [13-16].

The clinical relevance of these hemodynamic responses over the long term remains an open question. Episodic BP elevations following nicotine administration are a recognized pharmacological effect and are not equivalent to chronic hypertension [5,15]. Whether sustained daily ENDS use accumulates sufficient hemodynamic loading to drive left ventricular hypertrophy, aortic remodeling, or progressive arterial stiffening over years of exposure represents an important but currently unanswered question.

Endothelial function and arterial stiffness

Reductions in flow-mediated dilation (FMD) are the most mechanistically significant findings in trial literature. Nicotine-containing e-cigarettes reduce brachial artery FMD relative to sham conditions, with mean reductions in the range of 2 to 2.5 percentage points [17]. In general population studies, each 1% reduction in FMD has been associated with approximately a 13% increase in future cardiovascular event risk in population studies, although this relationship is derived from chronic baseline differences and may not apply to transient acute changes. These represent transient responses to a single-use session, not measures of sustained endothelial impairment [16,17]. Whether repeated daily exposure produces cumulative endothelial dysfunction that persists between sessions and ultimately contributes to structural vascular disease is not answered by the available trial evidence.

Arterial stiffness follows a comparable pattern. Acute e-cigarette use elevates PWV by approximately 0.9 m/s above sham on average, while IQOS produces comparable increases in the 0.7 to 0.9 m/s range [13,17]. For both outcomes, prediction intervals in pooled analyses are wide and include zero, underscoring the extent of between-study variability and the inadvisability of treating any single mean estimate as a reliable individual predictor.

Platelet activation

Platelet aggregometry and total thrombus area analyses demonstrate significant increases in platelet activation following nicotine-containing e-cigarette use, with standardized mean differences in the 0.6 to 0.8 range across available trials [17]. Elevated P-selectin, a marker of platelet and endothelial perturbation, is reported across multiple studies. IQOS produces broadly comparable effects. These thrombogenic signals may be the most clinically relevant acute finding, playing a major role in MI and coronary thrombosis [6]. The established caveat is that a prothrombotic response within a single exposure session does not determine whether sustained daily use maintains a chronically elevated thrombogenic state.

Inflammatory markers

Chronic inflammatory markers derived from cohort studies, mainly C-reactive protein (CRP) and interleukin-6 (IL-6), present a more equivocal picture. Some analyses report modest CRP elevations among e-cigarette users (standardized mean differences approximating 0.3), while IL-6 associations are weaker and inconsistent across studies [18]. Between-study variability is significant, confounding adjustment is rarely adequate, and sample sizes in well-adjusted analyses tend to be small. The hypothesis that chronic low-grade inflammation represents a pathway through which sustained ENDS use contributes to CVR is biologically plausible [18,19]. The observational evidence supporting this hypothesis is, at present, hypothesis-generating rather than confirmatory.

Biological mechanisms

The mechanistic evidence is more developed than the epidemiological evidence, given that experimental work in cell and animal models can establish causal relationships that observational studies in human populations are poorly positioned to isolate. Biological plausibility is not equivalent to established population-level risk.

The most characterized pathway involves oxidative stress and endothelial dysfunction. Nicotine, acrolein, formaldehyde, and metallic nanoparticles present in ENDS aerosol have been shown in experimental models to activate the NADPH oxidase 2 (NOX-2) system, generating reactive oxygen species that deplete endothelial nitric oxide through eNOS uncoupling and direct chemical quenching [8]. The resulting impairment of endothelium-dependent vasodilation could, over time, contribute to atherogenesis and adverse vascular remodeling. The acute FMD reductions observed in experimental studies are consistent with this mechanism operating over short timescales; whether it persists and accumulates with repeated exposure remains the unresolved question [20].

Nicotine mediates most of the downstream pharmacological activity. Through adrenergic receptor activation, it acutely raises HR, BP, and myocardial oxygen demand [13]. Through effects on thromboxane A2 and prostacyclin pathways, it shifts hemostatic balance toward a prothrombotic state [6]. Dose-response data from epidemiological subgroup analyses, with an MACE OR of approximately 1.6 for nicotine-containing devices compared with approximately 1.1 (non-significant) for nicotine-free products [4], support nicotine as a likely major contributor to the cardiovascular associations observed.

Other aerosol components carry independent vascular toxicity. Acrolein, present in both e-cigarette vapor and HTP aerosol, injures endothelium and promotes platelet activation through mechanisms that are at least partially independent of nicotine [21]. Carbonyl compounds impair cardiac mitochondrial function in cell and animal models [6,21]. Fine particulate matter shares certain pathways with air pollution, which is established as a CVR factor at the population level, though the relevant concentrations differ between these exposures [20]. HTPs occupy an intermediate mechanistic position. The elimination of full combustion reduces carbon monoxide, benzene, and polycyclic aromatic hydrocarbons, compounds that contribute to conventional tobacco's cardiovascular toll [19,20]. However, nicotine delivery is retained, carbonyl compounds are generated during the heating process, and tobacco-specific nitrosamines remain present [15,18]. The net effect of this altered toxicant profile on long-term CVR is not established.

Adolescents

E-cigarettes have become the predominant nicotine product among young people in several high-income countries [22,23]. There are biological grounds for hypothesizing that developing cardiovascular systems may be more susceptible to nicotine-induced perturbation than adult systems; however, this remains speculative. The epidemiological evidence concerning adolescents and cardiovascular outcomes is cross-sectional, involves small samples, and is limited by poor characterization of smoking history [20-23].

Nicotine content

The dose-response pattern for nicotine content is the most consistent finding across the ENDS cardiovascular literature. Cohort subgroup analyses demonstrate elevated MACE risk for nicotine-containing devices (OR approximately 1.6) relative to nicotine-free products (OR approximately 1.1, non-significant), with a statistically significant interaction term [4]. Experimental studies confirm that hemodynamic, vascular, and thrombogenic effects are largely or entirely nicotine-mediated [19]. The practical implication is that nicotine concentration and delivery efficiency are likely the most relevant product-level characteristics for CVR stratification, both in research design and in regulatory discussions regarding product standards [23].

Taken together, the evidence base is internally consistent but limited in interpretability: observational studies suggest modest elevations in cardiovascular risk, while experimental data demonstrate acute physiological effects consistent with vascular injury. However, substantial confounding by prior smoking and the absence of long-term data in never-smokers preclude confident estimation of the independent cardiovascular effects of ENDS.

Limitations

The current evidence on the cardiovascular effects of e-cigarettes is marked by several major uncertainties and limitations. It remains unclear how much of the observed risk is truly caused by e-cigarette use itself versus prior smoking history, and existing data do not allow firm conclusions in either direction. A key issue is the wide variation in what counts as e-cigarette use, ranging from occasional experimentation to heavy daily use of high-nicotine devices, with most studies failing to capture this complexity, leading to underestimation of risks for more intensive users. Experimental studies are also limited by small sample sizes, short-term designs, and a focus on young, healthy individuals, making it difficult to generalize findings or assess long-term effects. While short-term physiological changes from e-cigarette use are measurable and potentially harmful, there is no clear evidence yet that these translate into increased CVD over time, representing a central gap in the research. Much of the available data comes from outdated device types and populations in high-income regions, leaving important questions about current products and global patterns of use unanswered. 

A large portion of the available literature is cross-sectional in design, limiting causal inference. These studies often report larger effect estimates than prospective cohorts, a pattern consistent with reverse causation, whereby individuals with existing cardiovascular disease may transition from cigarettes to e-cigarettes. As exposure and outcome are measured simultaneously, these findings should not be used to estimate causal cardiovascular risk.

Clinical implications

ENDS use is not cardiovascularly neutral. Acute hemodynamic, endothelial, and thrombogenic effects are demonstrated in well-controlled experimental studies, and observational data, notwithstanding their limitations, are directionally consistent with modestly elevated long-term CVR relative to never-use [18-20]. These findings justify a clinical conversation with patients, particularly those who already carry CVR on account of established disease, hypertension, or arrhythmia, in whom the acute hemodynamic effects of nicotine-containing ENDS may have immediate clinical relevance [24]. For established smokers using ENDS as a cessation tool, the available data point in a favorable direction for those who achieve complete switching, suggesting lower CVR than continued exclusive smoking [22,23]. However, the certainty of this finding is low. Partial substitution of cigarettes with ENDS does not demonstrate a clear cardiovascular benefit over continued smoking and should not be presented to patients as a step toward harm reduction [19].

Nicotine content and delivery efficiency appear to be the most relevant product-level characteristics for cardiovascular risk, on the basis of subgroup data, though the evidence is not yet sufficient to support specific quantitative recommendations for individual patient counseling [25,26]. ENDS are not equivalent to conventional cigarettes, but they are not cardiovascularly safe [25]. Policy frameworks that distinguish combustible tobacco, ENDS, and non-nicotine products on a risk-stratified basis have some support in the available data but should be calibrated to reflect the low-to-very-low certainty of the underlying evidence rather than treating current estimates as more definitive than they are.

Research priorities

The most consequential gap in this literature is not methodological refinement, but the absence of large-scale prospective cohort studies enrolling verified never-smoking ENDS initiators with sufficient follow-up to detect hard cardiovascular endpoints. Without this design, the question of whether ENDS use independently causes CVD in individuals who have never smoked cannot be answered. Existing datasets that include detailed smoking history information could be reanalyzed with appropriate stratification to yield improved interim estimates. The designation "e-cigarette user" is scientifically imprecise. Standardized protocols for characterizing device type, nicotine concentration and delivery, frequency and duration of use, and inhalation topography, analogous to the pack-year metric in conventional tobacco research, would improve the interpretability and comparability of future studies.

For HTPs, independently funded prospective studies with longer follow-up and improved smoking history characterization represent the primary research priority. The current evidence base is dominated by industry-sponsored, short-duration research, which is an insufficient foundation for confident risk assessment.

## Conclusions

E-cigarette use appears to carry a modest increase in CVR compared to never-use, though much of this is likely influenced by prior smoking, making the true independent effect uncertain and probably smaller. Dual use is harmful, with significantly higher cardiovascular risk, and simply reducing cigarette consumption by adding e-cigarettes does not show a significant health benefit.

Comparisons with traditional cigarettes remain inconclusive, with only tentative signs that complete switching might reduce harm. The biggest gap is the lack of long-term data in people who have never smoked but use e-cigarettes. Both clinical guidance and policy should reflect this uncertainty rather than overstate the evidence.
